# Diagnosis of Sports-Related Concussion Using Symptom Report or Standardized Assessment of Concussion

**DOI:** 10.1001/jamanetworkopen.2024.16223

**Published:** 2024-06-11

**Authors:** Kimberly G. Harmon, Bridget M. Whelan, Douglas F. Aukerman, Calvin E. Hwang, Sourav K. Poddar, Anna DeLeo, Heather A. Elkington, Gabrielle Garruppo, Marissa Holliday, Jared M. Bruce

**Affiliations:** 1 Department of Family Medicine, University of Washington, Seattle; 2 Oregon State University, Corvallis; 3 Department of Orthopedic Surgery, Stanford University, Stanford, California; 4 Department of Family Medicine, University of Colorado, Boulder; 5 University of Washington, Seattle; 6 University of Colorado, Boulder; 7 Department of Biomedical and Health Informatics, University of Missouri, Kansas City School of Medicine, Kansas City

## Abstract

**Importance:**

The Sports Concussion Assessment Tool-5 (SCAT5) has been recommended for concussion evaluation and utilizes both a subjective reported symptom grading scale and objective measures of concussion including a cognitive evaluation: the Standardized Assessment of Concussion (SAC). The SAC includes testing for orientation, immediate memory, concentration, and delayed recall; a 10-word list is used to assess immediate memory and delayed recall.

**Objective:**

To determine the diagnostic accuracy of components of the SCAT5 and to provide a framework for clinical interpretation.

**Design, Setting, and Participants:**

This prospective case-control study of National Collegiate Athletic Association Division I athletes from any sport was conducted from July 2020 to December 2022 at 4 universities. Athletes completed baseline SCAT5 testing using the 10-word list. When an athlete presented acutely with suspected concussion (sideline or within 2 days), the tests were repeated. If a concussion was diagnosed, a control athlete underwent the same tests. Controls were identified and matched on comorbid conditions, sex and gender, sport, season, and baseline scores. Data analysis was conducted from August to October 2023.

**Main Outcomes and Measures:**

The primary outcomes were area under the receiver operating characteristic curve (AUC), sensitivity, specificity, positive and negative predictive value, and test-retest reliability of the symptom score; symptom severity score; the total SAC score; and the orientation, immediate memory, concentration, and delayed memory subcomponent scores on the SCAT5 compared with clinical diagnosis of concussion.

**Results:**

Baseline and postinjury data were collected on 92 athletes with concussion and 92 matched control athletes (96 men [52%] and 88 women [48%]; 110 who played a sport other than football [59%]). Diagnostic utility was considered excellent for symptom score (AUC, 0.93; 95% CI, 0.89-0.96) and symptom severity score (AUC, 0.94; 95% CI, 0.90-0.97). An increase of 2 points on the symptom score was associated with a sensitivity of 86% (95% CI, 78%-92%), specificity of 80% (95% CI, 70%-87I%), and positive predictive value of 81% (95% CI, 72%-88%). The total SAC score had poor to fair diagnostic utility (AUC, 0.70; 95% CI, 0.63-0.77); however, 41 athletes with concussion (45%) had a total SAC score at or above their baseline score (ie, within normal limits). The diagnostic utility was poor to fair for immediate memory (AUC, 0.68, 95%CI, 0.61-0.75) and delayed recall (AUC, 0.69; 95% CI, 0.62-0.77) and not useful for orientation (AUC, 0.49; 95% CI, 0.43-0.56) and concentration (AUC, 0.52 95% CI, 0.44-0.61). Test-retest reliability was fair for total SAC and poor for immediate memory and delayed recall, orientation, and concentration.

**Conclusions and Relevance:**

In this case-control study of the diagnostic accuracy of reported symptoms and the SAC, reported symptoms were the most accurate indicator of concussion while the 10-word SAC had limited sensitivity. These findings suggest that understanding the properties of the SAC is important when making the diagnosis of concussion.

## Key Points

**Question:**

Can symptom report and cognitive testing using the 10-word list of the Standardized Assessment of Concussion (SAC) be used to accurately diagnose concussion?

**Findings:**

In this case-control study of 184 participants assessing the diagnostic accuracy of reported symptoms and the SAC, an increase in symptom severity after suspected concussion was sensitive and specific for the diagnosis of concussion but was subjective. Objective cognitive testing using the SAC was not sensitive, and 41 athletes with concussion (45%) scored the same as their baseline score or better; the lower the SAC score below baseline, the more likely the athlete had a concussion.

**Meaning:**

These findings suggest that without an increase in reported symptoms, the SAC has limited ability to detect concussion.

## Teaser

This case-control study analyzes the diagnostic accuracy of various components of a concussion assessment that uses both patient report and standardized assessment among college athletes with and without concussion.

## Introduction

The sideline or acute diagnosis of concussion is often challenging, putting an athlete’s ability to return to competition on the line. There is currently no objective biomarker for concussion, and diagnosis relies heavily on athlete symptom reporting.^1^ The Sport Concussion Assessment Tool (SCAT) was introduced in 2004 by the Concussion in Sport Group^2^ as a standardized concussion evaluation combining several existing tools, including symptom evaluation (Post-Concussion Symptom Scale [PCSS]),^3^ cognitive evaluation (Standardized Assessment of Concussion [SAC]),^4^ balance assessment (Modified Balance Error Scoring System),^5^ and a neurological screening. Prior to the introduction of the SCAT, the evaluation of concussion was heterogeneous. This initial framework for evaluation has served as a foundation for improvements over the years.^6,7,8,9^

While not billed as a diagnostic tool, clinicians use the SCAT as part of a comprehensive clinical assessment that informs diagnosis. Consequently, it is important to understand the psychometric properties of the individual tests used to inform clinical decision-making. Previous studies^10,11^ show reported symptoms to be the most sensitive and specific measure for diagnosing concussion. However, relying on symptoms alone for concussion diagnosis can be problematic. Symptom reporting requires accurate disclosure by athletes who may be reluctant to report symptoms due to internal or external pressures, an inability to recognize symptoms, or a delay in the development of symptoms.^12^ The SAC is an objective measure of cognition that is included in the SCAT. The SAC is comprised of 4 subcomponents: (1) orientation, (2) immediate memory, (3) concentration, and (4) delayed recall. The original versions of the SAC used a 5-word list to test immediate memory and delayed recall.^13^ However, the 5-word list was criticized for having low sensitivity and specificity.^1,10,11,14,15,16,17,18^ The SCAT5 included the option to use a 10-word memory list, with hopes of improving utility and eliminating the ceiling effect.^19^ Studies examining diagnostic accuracy using the 10-word memory lists are limited. One study of professional hockey players using the 10-word list^10^ showed group differences between players with and without concussion, but the authors concluded the 10-word SAC had insufficient sensitivity and specificity to be used as a stand-alone diagnostic tool.

Clinicians interpreting SCAT scores can use either normative data or change scores derived from a previously performed baseline test. SCAT normative values may differ based on the age and sex of the athlete.^20,21,22,23^ Consequently, normative approaches require familiarity with typical scores obtained in the population being evaluated. This approach can result in the misdiagnosis of people who would naturally score either very high or very low on the SCAT, even when not concussed. This issue can be overcome by comparing test scores obtained following a suspected concussion to baseline scores previously obtained. The purpose of this study was to assess the diagnostic accuracy of the PCSS and the 10-word SAC using a baseline-retest approach.

## Methods

This case-control study was approved by the University of Washington Human Subjects Division with reliance agreements from other institutions and followed the Strengthening the Reporting of Observational Studies in Epidemiology (STROBE) reporting guideline. English-speaking National Collegiate Athletic Association (NCAA) athletes in any sport from 4 institutions (University of Washington, Oregon State University, Stanford University, and University of Colorado) were eligible for inclusion if they sustained a concussion from July 13, 2020, to December 31, 2022, and had an acute concussion evaluation within 48 hours of injury. Athletes were excluded if they had a concussion in the previous year and if they did not have a complete baseline and acute injury evaluation using the 10-word memory list for both the athlete with concussion and the control athlete. All athletes completed electronic informed consent. All athletes had complete baseline concussion testing done prior to participating in sport at least once as required by the NCAA. Baseline frequency was determined by the practice of the university, which often differed by sport. In most contact sports, baseline testing was performed annually. If an athlete was concussed and agreed to participate in the study, a matched control athlete was selected and invited to participate based on team, sex and gender, concussion comorbidities (clinically diagnosed attention-deficit/hyperactivity disorder [ADHD], learning disorder, migraine headache disorder, or mood disorder) and baseline total symptom and total SAC scores within a 2-point range. The control athlete was given the SCAT5 within 2 weeks of the incident concussion, so season and school stressors were similar to those of the athlete with concussion. This also meant the athlete with concussion and the control athlete had a similar amount of time between initial baseline testing (which is done at the same time for teams) and repeat testing. The diagnosis of concussion was made by team medical personnel using the definition from the Concussion in Sports Group.^24^ The PCSS and SAC with the 10-word memory list were performed by team medical staff as described in the SCAT5.^25^ A total score was calculated for the SAC, which included orientation (0-5 points), immediate memory (0-30 points), concentration (0-5 points), and delayed recall (0-10 points), with a maximum of 50 points possible. Higher symptom scores and symptom severity scores indicate worse symptoms while higher scores on the total SAC and its subcomponents indicate better performance. Time from injury to the SAC evaluation was also noted as immediate (≤2 hours), between 2 and 8 hours, between 8 and 24 hours, and between 24 and 48 hours. Self-identified race and ethnicity was recorded to ensure the sample was comparable with the race and ethnicity distribution of NCAA athletes. Race and ethnicity groups included African American or Black, Asian, Hispanic or Latinx, Native Hawaiian or Pacific Islander, White, and multiracial.

### Statistical Analysis

Normality of all variables was assessed using Shapiro-Wilk test and χ^2^ analyses were used to examine between group differences in gender, sport, year in school, and comorbidities. Differences within and between groups and time points were assessed using 2 sample *t* tests of unequal variances (Welch *t* test) and the Mann-Whitney *U* test. Test-retest reliability from baseline to control follow-up was assessed using 2-way intraclass correlations using absolute agreement for the symptom score, symptom severity score, and the total score for the 10-word SAC and subcomponents.^26^ Sensitivity, specificity, positive predictive value (PPV), and negative predictive value (NPV) were calculated for each testing component at various cut points. We examined the role of possible covariates (eg, ADHD diagnosis, migraine disorder diagnosis, mood disorder, and year in school) using logistic regression. The covariates were identified a priori based on previous literature and clinical expertise.^8,27^ None were significant and were subsequently excluded from the models. A model was built using logistic regression to examine the association of symptom score, symptom severity score, and total SAC score with the outcome. Logistic regression was used to address any violations of the assumptions of independence, homoscedasticity, and normality. Given the binary nature of the outcome variable (presence or absence of concussion) and a violation of normality in some of the data, we opted for a binomial distribution and specified a logit link function to model the association. Receiver operating characteristic curves (ROC), area under the ROC curve (AUC), and 95% CIs were calculated to assess test validity for all postinjury assessments; excellent diagnostic utility was considered to be an AUC between 0.9 and 1.0; good diagnostic utility, between 0.8 to less than .09; fair diagnostic utitlity, between .07 to less than .08; and poor diagnostic utility, less than .0.6. All analyses were examined at an α level of .05.

Analyses were conducted in RStudio version 2023.06.1 + 524 (Posit) using the dplyr, ggplot, irr, and pROC packages. Data analysis occurred from August 2023 to October 2023.

## Results

### Demographics

There were 100 athletes with concussion and 100 control athletes initially enrolled. Eight pairs were dropped because either the athlete with concussion or the control athlete did not have complete information recorded, resulting in a final analytical sample of 184 athletes (96 men [52%] and 88 women [48%]; 15 African American or Black [8%]; 7 Asian [4%]; 6 Hispanic or Latinx [3%]; 9 Native Hawaiian or Pacific Islander [5%]; 107 White [58%]; 35 multiracial [19%]). There were 92 concussions that met inclusion criteria diagnosed during the study period. The distributions of gender and sex, and race and ethnicity closely mirrored that of the larger population of NCAA athletes.^28^ Of all athletes, 110 (59%) played a sport other than football. Concussions occurred most often in football (37 concussions [40%]) and volleyball (14 concussions [15%]). Of all participants, 6 (3%) had diagnoses of ADHD, 43 (23%) had mood disorders (anxiety or depression), and 14 (8%) had headache or migraine disorder; no participants had a learning disability. Concussions occurred most often in the second year of school. There were no significant differences between athletes with concussion and control athletes (Table 1).

**Table 1.** Demographics^a^

Characteristic	Participants, No (%)		
	Total (N = 184)	Concussion (n = 92)	Control (n = 92)
Gender			
Men	96 (52)	48 (52)	48 (52)
Women	88 (48)	44 (48)	44 (48)
Sport			
Men’s Football	74 (40)	37 (40)	37 (40)
Women’s Volleyball	26 (14)	14 (15)	12 (13)
Women’s Softball	17 (9)	7 (8)	10 (11)
Women’s soccer	13 (7)	1 (1)	6 (6)
Men’s Baseball	11 (6)	5 (5)	6 (6)
Women’s basketball	7 (4)	3 (3)	4 (4)
Women’s track and field	7 (4)	7 (8)	4 (4)
Men’s rowing	6 (3)	3 (3)	3 (3)
Women’s rowing	6 (3)	3 (3)	3 (3)
Women’s Beach volleyball	4 (2)	2 (2)	2 (2)
Men’s soccer	4 (2)	2 (2)	2 (2)
Cheer (men’s and women’s combined)	3 (2)	2 (2)	1 (1)
Women’s Gymnastics	2 (1)	1 (1)	1 (1)
Women’s tennis	2 (1)	3 (3)	1 (1)
Men’s basketball	1 (1)	1 (1)	0
Women’s golf	1 (1)	1 (1)	0
Year in School			
1	48 (26)	25 (27)	23 (25)
2	67 (36)	31 (33)	36 (39)
3	35 (19)	19 (21)	16 (17)
4	28 (15)	15 (16)	13 (14)
≥5	6 (3)	2 (2)	4 (4)
Comorbidities			
Attention-deficit/hyperactivity disorder	6 (3)	4 (4)	2 (2)
Headache or migraine	14 (8)	7 (8)	7 (8)
Learning disorder	0	0	0
Mood disorder (anxiety and/or depression)	43 (23)	21 (23)	22 (24)

^a^ χ^2^ analysis found no differences between concussion and control groups.

### Reliability

The median (IQR) number of days between the baseline and the repeat test was 147 (86-125) days for the symptom score and 198 (117-420) days for the SAC. Median (IQR) number of days between baseline and repeat test for SAC were similar in both athletes with concussion (188 [11-114] days) and control athletes (212 [130-421] days). All tests had poor test-retest reliability, meaning there was significant variation in testing from test to test even in athletes without a concussion. (Table 2).

**Table 2.** Test-Retest Reliability Using ICC

Test	ICC (95% CI)	*P* value
Symptom score	0.57 (0.42 to 0.70)	<.001
Symptom severity	0.60 (0.45 to 0.72)	<.001
Total Standardized Assessment of Concussion	0.29 (0.09 to 0.47)	.002
Orientation	0.17 (−0.04 to 0.37)	.06
Immediate memory	0.19 (−0.01 to 0.37)	.03
Concentration	0.43 (0.25 to 0.59)	<.001
Delayed recall	0.24 (0.04 to 0.43)	.01

Abbreviation: ICC, intraclass correlation coefficient.

### Baseline Tests

The median (IQR) baseline scores for all athletes using the 10-word memory list was 37.5 of 50.0 (43.0-40.0) for total SAC, orientation 5.0 of 5.0 (5.0-5.0) for orientation, 21.0 of 30.0 (20.0-23.0) for immediate memory, concentration 4.0 of 5.0 (3.0-5.0) for concentration, and 7.0 of 10.0 (6.0-9.0) for delayed recall. The total SAC score was normally distributed. There were no significant differences between baseline scores between the concussion and control groups (Table 3).

**Table 3.** Baseline and Postinjury Time Point Values for Concussion and Control Groups

Test	Concussion baseline	Control baseline	*P* value^a^	Concussion postinjury	Control postinjury time point	*P* value^a^
Symptom score						
Mean (SD)	2.20 (3.12)	1.79 (3.09)	.20	11.85 (5.81)	2.11 (3.29)	<.001^b^
Median (range)	1.0 (0.0-15.0)	0.0 (0.0-16.0)		11.0 (0.0-22.0)	0.0 (0.0-18.0)	
Change in mean score from baseline, No. of points	NA	NA	NA	9.65	0.36	
Symptom severity						
Mean (SD)	3.82 (6.43)	2.71 (6.11)	.11	29.96 (21.86)	3.08 (5.49)	<.001^b^
Median (range)	1.0 (0.0-37.0)	0.0 (0.0-45.0)		24.5 (0.0-93.0)	0.0 (0.0-33.0)	
Change in mean score from baseline, No. of points	NA	NA	NA	26.14	0.42	
Total Standardized Assessment of Concussion score						
Mean (SD)	37.30 (4.67)	37.35 (3.48)	.97^c^	35.09 (5.99)	39.33 (4.38)	<.001^b^^,^^c^
Median (range)	37.5 (27.0-46.0)	37.5 (27.0-47.0)		35.0 (20.0-47.0)	40.0 (26.0-49.0)	
Change in mean score from baseline, No. of points	NA	NA	NA	−2.2X	1.98	
Orientation						
Mean (SD)	4.89 (0.31)	4.90 (0.30)	.07	4.72 (0.60)	4.82 (0.39)	.04^b^
Median (range)	5.0 (4.0-5.0)	5.0 (4.0-5.0)		5.0 (2.0-5.0)	5.0 (3.0-5.0)	
Change in mean score from baseline, No. of points	NA	NA	NA	−0.17	−0.02	
Immediate memory						
Mean (SD)	21.51 (3.11)	21.21 (2.45)	.39	20.32 (3.80)	22.85 (2.85)	<.001^b^
Median (range)	22.0 (14.0-27.0)	21.0 (13.0-27.0)		20.5 (14.0-29.0)	23.0 (14.0-29.0)	
Change in mean score from baseline, No. of points	NA	NA	NA	−1.19	1.51	
Concentration						
Mean (SD)	3.64 (1.09)	3.82 (1.14)	.22	3.76 (1.09)	3.92 (1.09)	.31
Median (range)	4.0 (1.0-5.0)	4.0 (1.0-5.0)		4.0 (1.0-5.0)	4.0 (1.0-5.0)	
Change in mean score from baseline, No. of points	NA	NA	NA	0.17	0.16	
Delayed recall						
Mean (SD)	7.31 (1.73)	7.37 (1.50)	.98	6.23 (2.27)	7.73 (1.70)	<.001^b^
Median (range)	8.0 (3.0-10.0)	7.0 (4.0-10.0)		6.0 (0.0-10.0)	8.0 (0.0-10.0)	
Change in mean score from baseline, No. of points	NA	NA	NA	−1.08	0.30	

Abbreviation: NA, not applicable.

^a^ Mann-Whitney *U* test unless otherwise specified.

^b^ Significant.

^c^ Welch *t* test.

### Diagnostic Accuracy

In athletes with concussion, mean scores increased (ie, worsened) a total of 9.65 points for the symptom score (mean [SD] score, 2.20 [3.12] at baseline vs 11.85 [5.81] at postinjury; *P* < .001) and 26.14 points for symptom severity score (mean [SD] score, 3.82 [6.43] at baseline vs 29.96 [21.86] at postinjury; *P* < .001). The mean total SAC score in the group of athletes with concussion decreased (ie, worsened) by 2.21 points (mean [SD] score, 37.30 [4.67] at baseline vs 35.09 [5.99] at postinjury; *P* = .01), orientation decreased by 0.17 points (mean [SD] score, 4.89 [0.31] at baseline vs 4.72 [0.60] postinjury; *P* = .04), immediate memory decreased by 1.19 points (mean [SD] score, 21.51 [3.11] at baseline vs 20.32 [3.80] at postinjury; *P* < .001 ), and delayed recall decreased by 1.08 points (mean [SD] score, 7.31 [1.73] at baseline vs 6.23 [2.27] postinjury; *P* < .001). In control athletes retaking the test, symptom score and symptom severity score did not significantly change, however the mean total SAC score increased (ie, improved) by 1.98 points (mean [SD] score, 37.35 [3.48] at baseline vs 39.33 [4.38] at control postinjury time point; *P* < .001) and the mean immediate memory score increased by 1.51 points (mean [SD] score, 21.21 [2.45] at baseline vs 22.85 [2.85] at control postinjury time point; *P* < .001) (Table 3).

Symptom score, symptom severity score, total SAC score, immediate memory score, and delayed recall score differentiated the groups of athletes with and without concussion. However, in individual athletes, only symptom score (AUC, 0.93; 95% CI, 0.89 to 0.96) and symptom severity score (AUC, 0.94; 95% CI, 0.90 to 0.97) had excellent diagnostic utility (as defined in the Methods) (Table 4). Tests with poor to fair utility included the total SAC score (AUC, 0.70; 95% CI, 0.63 to 0.77), immediate memory (AUC, 0.68; 95% CI, 0.61 to 0.75), and delayed recall (AUC, 0.69; 95% CI, 0.62 to 0.77). Tests with little to no diagnostic utility included for orientation (AUC, 0.49; 95% CI, 0.43 to 0.56) and concentration (AUC, 0.52; 95% CI, 0.44 to 0.61) with 95% CIs crossing over 0.50 (Table 4). The total SAC, immediate memory, and delayed recall were poorly sensitive but more specific as the cut point increased. The sensitivity, specificity, PPV, and NPV are shown for symptom score, symptom severity score, total SAC, orientation, immediate memory, concentration, and delayed recall (Table 4). The cut point data show that an increase of 2 points on the symptom score was associated with a sensitivity of 86% (95% CI, 78% to 92%), specificity of 80% (95% CI, 70% to 87%), and PPV of 81% (95% CI, 72% to 88%). An increase of either symptom score or symptom severity score by 8 points had 100% PPV. A decline of 3 points on delayed recall had limited sensitivity (26%; 95% CI, 18%-36%) but high specificity (96%; 95% CI, 89% to 99%). Likewise, a 6-point decrease on immediate memory had only 13% sensitivity for concussion (95% CI, 7% to 22%) but was 98% specific (95% CI, 92% to 100%). Of note, 41 athletes with concussion (45%) scored the same or better than their baseline SAC score (Table 4). ROC curves are presented in the Figure. A logistic regression model was used to determine if the total SAC score added additional diagnostic value above the symptom score alone. The model showed that both total SAC (β=−0.12; 95% CI, −0.21 to −0.03; *P* = .01) and symptoms scores (β=0.41; 95% CI, 0.30 to 0.53; *P* < .001) accounted for unique variance in concussion diagnosis. The postinjury symptom score was (AUC, 0.93; 95% CI, 0.89 to 0.96) and symptom score and total SAC score combined (AUC, 0.94; 95% CI, 0.90 to 0.97) indicated excellent diagnostic utility (Figure, D).

**Table 4.** Sensitivity, Specificity, PPV, NPV, and AUC of Symptoms and Total SAC Score and Subcomponent Scores

Scale^a^	Sensitivity, % (95% CI)^b^	Specificity, % (95% CI)^c^	PPV, % (95% CI)^d^	NPV, % (95% CI)^e^	AUC (95% CI)
Symptoms score increase, points					
1	88 (80-94)	73 (63-81)	75 (66-83)	87 (78-93)	0.93 (0.89-0.96)
2	86 (78-92)	80 (70-87)	81 (72-88)	85 (78-93)	
3	81 (72-89)	82 (73-89)	82 (73-89)	81 (76-92)	
4	77 (67-85)	86 (78-93)	85 (76-92)	79 (72-89)	
5	72 (62-81)	90 (82-95)	89 (79-95)	76 (70-86)	
6	68 (57-77)	92 (84-96)	89 (79-95)	74 (67-83)	
7	64 (53-73)	97 (91-99)	95 (87-99)	72 (65-82)	
8	57 (48-69)	100 (92-100)	100 (88-100)	68 (59-76)	
Symptom severity score increase, points					
1	90 (81-95)	72 (62-81)	72 (62-81)	90 (81-95)	0.94 (0.90-0.97)
2	87 (78-94)	79 (70-87)	78 (68-86)	88 (79-94)	
3	84 (74-91)	82 (72-89)	80 (69-88)	85 (76-92)	
4	79 (69-88)	86 (77-92)	83 (72-90)	83 (74-90)	
5	NA	90 (82-95)	NA	NA	
6	NA	91 (84-96)	NA	NA	
7	78 (67-87)	97 (91-99)	95 (88-99)	84 (75-90)	
8	77 (66-86)	NA	10 (94-100)	NA	
Total SAC score decrease, points					
1	55 (45-65)	75 (65-83)	68 (57-78)	63 (53-71)	0.70 (0.63-0.77)
2	49 (39-59)	81 (71-88)	73 (60-83)	60 (51-69)	
3	44 (34-55)	83 (74-90)	72 (59-83)	60 (51-68)	
4	40 (30-50)	86 (77-92)	75 (61-86)	58 (49-66)	
5	34 (25-48)	88 (80-94)	73 (58-85)	58 (50-66)	
6	29 (20-39)	95 (88-99)	85 (69-95)	55 (47-63)	
7	21 (13-30)	96 (89-99)	83 (63-95)	54 (46-62)	
8	17 (10-26)	97 (91-99)	84 (60-97)	54 (46-61)	
9	15 (8-24)	98 (93-100)	88 (62-98)	54 (46-61)	
Orientation score decrease, points					
1	15 (9-24)	91 (84-96)	65 (43-84)	51 (43-58)	0.49 (0.43-0.56)
2	3 (1-10)	99 (94-100)	75 (19-99)	50 (43-58)	
3	1 (0-6)	NA	100 (2-100)	NA	
Immediate memory score decrease, points					
1	50 (40-60)	77 (70-85)	70 (58-80)	59 (49-68)	0.68 (0.61-0.75)
2	40 (30-51)	82 (73-90)	70 (56-82)	58 (49-66)	
3	33 (24-44)	88 (79-94)	74 (58-86)	56 (47-64)	
4	26 (18-37)	91 (83-96)	75 (57-88)	54 (46-62)	
5	20 (13-30)	92 (85-97)	73 (52-88)	53 (45-61)	
6	13 (7-22)	98 (92-100)	86 (57-98)	52 (45-60)	
Concentration score decrease, points					
1	22 (14-32)	78 (69-85)	49 (34-64)	51 (43-59)	0.52 (0.44-0.61)
2	10 (5-18)	95 (88-98)	64 (35-87)	51 (43-58)	
3	2 (0-6)	NA	100 (2-100)	NA	
Delayed recall score decrease, points					
1	51 (41-61)	71 (61-79)	63 (51-74)	60 (50-69)	0.69 (0.62-0.77)
2	39 (29-49)	84 (75-90)	71 (57-82)	57 (49-66)	
3	26 (18-36)	96 (89-99)	86 (68-96)	54 (46-62)	
4	16 (9-25)	NA	100 (78-100)	NA	

Abbreviations: AUC, area under the receiver operator characteristic curve; NA, not applicable; NPV, negative predictive value; PPV, positive predictive value.

^a^ The symptoms scale score ranges from 0 to 22; symptom severity score, 0 to 132; SAC score, 0 to 50; orientation score, 0 to 5; immediate memory, 0 to 30; concentration, 0 to 5; and delayed recall, 0 to 10.

^b^ Probability of a positive test in those with a concussion.

^c^ Probability of a negative test in those without a concussion.

^d^ Probability of having a concussion if the test is positive.

^e^ Probability of not having a concusssion if the test is negative.

**Figure.**
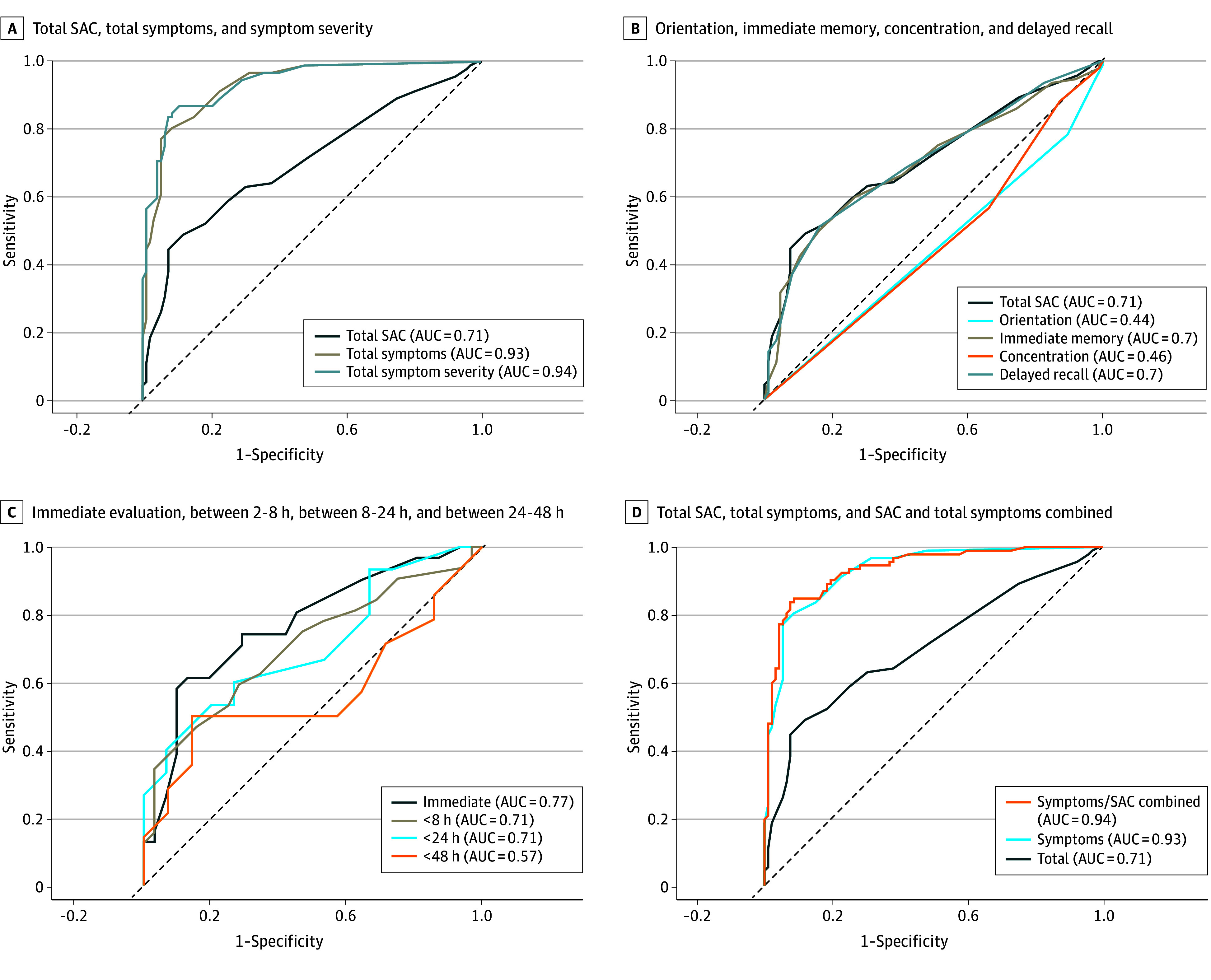
Receiver-Operator Characteristic Curves The figure shows receiver-operator characteristic curves for total SAC, total symptoms, and symptom severity (A); orientation, immediate memory, concentration, and delayed recall (B); immediate evaluation (≤2 hours), evaluation between 2 and 8 hours, evaluation between 8 and 24 hours, and evaluation between 24 and 48 hours (C); and total SAC, total symptoms, and total SAC + total symptoms (D). AUC indicates area under the receiver-operator characteristic curve; SAC, Standardized Assessment of Concussion.

### Time to Examination

Of the 92 participants with concussion, 31 (34%) were evaluated immediately (2 hours or less), 15 (16%) between 2 and 8 hours postinjury, 32 (35%) between 8 and 24 hours postinjury, and 14 (15%) between 24 and 48 hours postinjury. The diagnostic utility of the total SAC score decreased over time, with an AUC of 0.77 ( 95% CI, 0.64-0.88) for the immediate time point, 0.71 (95% CI, 0.53-0.86) for the 2- to 8-hour time point, 0.71 (95% CI, 0.58-0.83) for the 8- to 24-hour time point, and 0.57 (95% CI, 0.35-0.79) for the 24- to 48-hour time point; however, the sample of athletes in each group was small and 95% CIs overlapped (Figure, C).

## Discussion

This case-control study highlights several important points. First, the development of symptoms after a suspected concussive blow was the most accurate indicator of concussion with an AUC of 0.93 for symptom score and 0.94 for symptom severity score. Second, the sensitivity and specificity of the 10-word SAC for the diagnosis of concussion reached only fair accuracy (AUC, 0.71), although it was slightly improved compared with previously reported values for the 5-word SAC (AUC, 0.68).^14^ Importantly, almost one-half of concussed athletes (45%) performed at or above their baseline test results (ie, within normal limits) on the SAC, highlighting the limitations of the cognitive examination and the importance of an increase in symptoms, as well as including other objective indicators of concussion such as visual signs and vestibulo-ocular or balance abnormalities. Indeed, the AUC for the symptom score combined with the total SAC score (0.94) was only slightly higher than the AUC for the symptom score alone (0.93), indicating the total SAC adds minimal additional projected variance, which is likely not clinically significant. Third, there was considerable performance variability over time in the 10-word SAC in athletes without concussion. Fourth, the orientation and concentration subcomponents did not add to the diagnostic utility of the SAC, similar to results found in a sample of professional hockey athletes.^10^ Fifth, the accuracy of the SAC decreased as time from the incident increased, with an AUC of 0.77 at the immediate time frame and AUC of only 0.57 with confidence intervals crossing the 0.5 threshold after 24 hours, although these subsamples were small and the confidence intervals were large and overlapped.

In this sample of athletes with concussion, there was an increase of 9.65 points in mean symptom score from baseline score in athletes with concussion and an increase of 26.14 points in mean symptom severity score from baseline. The cut point data in Table 4 showed an increase of either symptom score or symptom severity score by 8 points had 100% PPV. In contrast, symptom score and symptom severity score did not increase significantly in the control athletes. Although an increase in symptoms is highly suggestive of concussion, this relies on accurate reporting by the athlete who may not report symptoms because of a desire to return to play, a fear of letting teammates down, minimizing the seriousness of concussion, difficulty discerning symptoms, a delay in symptom development, or other reasons. This finding highlights the importance of educating athletes and checking in after the initial evaluation. Although the sensitivity of the SAC was relatively low, when the SAC is below baseline, it can inform concussion diagnosis, primarily by changes in immediate memory and delayed list recall. For instance, while having limited sensitivity (26%), a decline of 3 points on the delayed recall portion of the SAC was highly specific (96%). Likewise, a 6-point decrease on immediate memory had only 13% sensitivity for concussion but was 98% specific. These results show that learning and memory assessment cannot be used in isolation to diagnose concussion; however, a substantial minority of athletes with concussion have memory problems that, when present, are highly suggestive of concussion.

Test-retest reliability is typically measured in weeks or months compared with the 147 days for symptoms and 198 days for the total SAC in this study. However, these longer time frames represent how the test is utilized in actual practice. NCAA athletes are required to have baseline testing performed at least once, but it is not always performed annually, particularly in sports where the risk for concussion is lower.^29^ Even when annual baseline cognitive testing is done, it may still be many months before an athlete with a suspected concussion has repeat testing. Annual baseline testing for all athletes requires a substantial amount of time and person-power and may not be possible at lower-resourced institutions or for secondary or youth sports. Understanding normative scores is important in these situations. Use of normative scores has been shown to be as accurate as using the baseline and postinjury repeat testing paradigm, but this may be because of suboptimal psychometrics when using both the baseline-retest and normative approaches.^10,18,30,31^

The accuracy of the SAC decreased as the time from the injury increased. Although the accuracy decreased, the number of athletes in each group was relatively small and the confidence intervals overlapped. A recent systematic review of the SCAT,^32^ which included reporting on 14 acceptable or high-quality studies on SAC score and timing of exam, noted that the greatest differences in standard mean difference of total 5-word SAC score was in the first 24 hours after injury, which is supported by our findings. Larger groups at different time points may help inform at what time point the SAC should no longer be used as an aid to diagnosis.

The decision to diagnose an athlete with a concussion is difficult, and medical clinicians must balance the possibilities of letting someone continue to play with concussion vs removing someone from play who does not have a concussion. The sensitivity, specificity, PPV, and NPV can all be used to help make these types of decisions. Clinicians may have different thresholds balancing these competing interests depending on the clinical situation, but Table 4 can provide data to inform decision-making. The worse an athlete scores when compared with their baseline score, the more likely they are to have a concussion. However, an athlete may test normally (or better than baseline) and still be concussed with elevated symptoms and/or ataxia or loss of consciousness.

Strengths of the study include a sample that is almost one-half women, a broad representation of sports, and a racial and ethnic distribution similar to that of NCAA athletes. The use of control athletes matched for ADHD, mood disorders, migraine or headache disorders, and learning disorders for repeat testing at a similar time of the school year to the athlete with concussion also represents a strength. The SCAT5 was used for this study, however a newer version, the SCAT6 was recently published.^33^ The SAC component of the SCAT6 is identical to the SCAT5 with the exception of the timing the months backward task in the concentration subcomponent.^34^ This task accounts for 1 point, and we do not believe that this change will significantly change our results.

### Limitations

There were limitations to the study. This study included Division I athletes and may not represent other populations such as high school or professional athletes. There are 3 different word lists available in the SAC to decrease the possibility of learning effect, however, a study in professional hockey players has shown that the 10-word lists are not equivalent.^35^ Nonequivalent lists may account for the SAC improvements observed in our control group upon retest. We did not control for list order in this study and do not believe that it is practical in most acute or sideline settings, especially in the community. Rather than controlling for list order, we would recommend creating equivalent lists in future iterations of the SCAT or development of automated regression-based norms that correct for list differences as part of an application-based delivery of the SCAT. We did not control for concussion history although we excluded athletes or controls with a concussion in the previous year. Studies have shown that those with more than 3 concussions may have lower baseline performance on the SAC.^23^

## Conclusions

This case-control study affirms that reported symptoms are the most sensitive indicator of concussion and there are limitations to the objective cognitive testing included in the SCAT. An athlete with a clear mechanism of injury who develops symptoms should be considered concussed unless there is a reasonable alternative explanation for these symptoms. The use of the 10-word SAC has eliminated the ceiling effect present with the 5-word SAC but has only minimally increased the accuracy from previously reported values when using the baseline-retest approach.^14^ The lack of utility of the orientation and concentration subcomponents should be considered in future versions of the SAC. In this study, 45% of athletes with a concussion scored at or above their baseline scores (ie, within normal limits), emphasizing the need for multimodal evaluation, including attention to symptoms, visible signs, and vestibulo-ocular components. In cases where the total SAC or any of the subcomponents are well below baseline, a diagnosis of concussion should be considered. The cut points provided in this study can be used to guide decision making; declines of 3 points or more on immediate memory or delayed recall or a decline of 6 points on the total SAC score should be considered concerning. More research into appropriate time frames for use of the SAC should be conducted. Concussion remains a clinical diagnosis that should be based on a thorough review of signs, symptoms, and clinical findings.

## Notes

Supplement.
Data Sharing Statement
