# Supplementary material for: Diagnosis of Sports-Related Concussion Using Symptom Report or Standardized Assessment of Concussion
Source: JAMA Netw Open. 2024 Jun 11;7(6):e2416223. doi: 10.1001/jamanetworkopen.2024.16223 (PMC11167498; doi:10.1001/jamanetworkopen.2024.16223)
Supplement: Supplement. — Data Sharing Statement [file jamanetwopen-e2416223-s001.pdf]

Data Sharing Statement

Harmon. Diagnosis of Sports-Related Concussion Using Symptom Report or Standardized Assessment of Concussion. *JAMA Netw Open*. Published June 11, 2024. doi:10.1001/jamanetworkopen.2024.16223

Data
Data available: No

Additional Information
Explanation for why data not available: Data use agreement with UW is needed
